# Polymyalgia Rheumatica Mimicking Infection and Malignancy: A Diagnostic Challenge Ruled Out by PET-CT

**DOI:** 10.7759/cureus.96072

**Published:** 2025-11-04

**Authors:** May Wathan Aung, Batsi Chikura, Sally Mubarak, Khin Lei Lei Aung, Farooq Ahmad, Htet Su Win

**Affiliations:** 1 General Internal Medicine, United Lincolnshire Hospitals NHS Trust, Lincoln, GBR; 2 Rheumatology, United Lincolnshire Hospitals NHS Trust, Lincoln, GBR; 3 Radiology, United Lincolnshire Hospitals NHS Trust, Lincoln, GBR; 4 Medicine, Grantham and District Hospital, Lincoln, GBR

**Keywords:** atypical presentation, differential diagnosis, infection, malignancy, pet-ct, polymyalgia rheumatica

## Abstract

Polymyalgia rheumatica (PMR) is an inflammatory disorder affecting older adults, typically presenting with pain and stiffness in the shoulder and pelvic girdle. The clinical features may overlap with infection or malignancy, often leading to delayed treatment and extensive, sometimes unnecessary, investigations. We present a case of an elderly patient with prolonged shoulder pain and elevated inflammatory markers who underwent a comprehensive evaluation. MRI of the shoulder and PET-CT were performed to exclude alternative diagnoses. The imaging findings, combined with the clinical presentation and a rapid response to corticosteroid therapy, confirmed the diagnosis of PMR. This case underscores the pivotal role of PET-CT in the diagnostic workup of atypical PMR, facilitating the exclusion of sinister pathologies such as malignancy or infection, guiding clinical decision-making, and reducing the need for unnecessary invasive investigations.

## Introduction

Polymyalgia rheumatica (PMR) is a common inflammatory disorder in older adults, frequently associated with giant cell arteritis, and typically presents with proximal muscle pain and stiffness [1]. Recent reviews have highlighted the epidemiology, pathophysiology, and management of PMR, emphasising the diagnostic challenges arising from its clinical overlap with other conditions [2]. Despite well-recognised features, diagnosis remains difficult because PMR can mimic systemic infections, malignancies, and other inflammatory diseases, often leading to delayed treatment and unnecessary investigations [3].

Imaging modalities such as ultrasonography and MRI can demonstrate bursitis and synovitis in the shoulders, providing supportive evidence in suspected cases [4]. More recently, PET-CT has gained recognition for its ability to detect bursitis, assess large-vessel involvement, and differentiate PMR from other serious conditions such as malignancy or infection [5].

## Case presentation

A 68-year-old man with a past medical history of hypertension and dyslipidemia presented with bilateral shoulder pain and morning stiffness, with each episode lasting more than two hours. The symptoms had persisted for several months and were associated with generalized fatigue and weight loss. He denied fever, night sweats, or recent infections.

On examination, he appeared tired but was afebrile. There was a restricted range of motion in both shoulders due to pain, with tenderness over the proximal muscles of the upper arms. No synovitis or joint swelling was observed; the system examination was otherwise unremarkable.

Initial laboratory investigations revealed markedly elevated inflammatory markers, with a CRP level of 85 mg/L (reference < 5 mg/L) and an ESR of 95 mm/hr (Table 1). White blood cell count, hemoglobin, and platelet counts were within normal limits. Renal and liver function tests were also normal. The myeloma screen was negative. Autoimmune screening, including antinuclear antibodies and rheumatoid factor, was negative. Repeated blood, urine, and stool cultures were negative.

**Table 1:** Initial laboratory results showing markedly elevated inflammatory markers (CRP 294 mg/L, ESR > 140 mm/hr), thrombocytosis, hypoalbuminemia, and raised ferritin, consistent with a systemic inflammatory process. Autoimmune and infectious screens were negative.

Blood Test	Test value	Normal value	
Hemoglobin	116	132-170 g/L	
White cell count	12.1	4.3-11.2 x10⁹/L	
Platelets	630	150-400 x10⁹/L	
Red cell count	3.95	4.29-5.69 x10¹²/L	
Mean cell volume	85	81-97 fL	
Hematocrit	0.337	0.387-0.492	
Mean corpuscular hemoglobin	29.4	26.9-33 pg	
Mean corpuscular hemoglobin concentration	344	320-359 g/L	
Neutrophils	9.28	2.1-7.4 x10⁹/L	
Lymphocytes	1.26	1.0-3.6 x10⁹/L	
Monocytes	1.43	0.3-1.0 x10⁹/L	
Eosinophils	0.17	0.02-0.5 x10⁹/L	
Basophils	0.05	0.02-0.1 x10⁹/L	
ESR	>140	5-20 mm/h	
CRP	294	0-5 mg/L	
Vitamin B12	367	197-771 ng/L	
Serum folate	1.4	2.0-18.7 µg/L	
Ferritin	1058	40-405 µg/L	
Serum iron	2.4	5.83-34.5 µmol/L	
Total iron binding capacity	28	45-70 µmol/L	
Transferrin	1.13	2-3.6 g/L	
Transferrin saturation	8	20-55 %	
Sodium	136	133-146 mmol/L	
Potassium	4.2	3.5-5.3 mmol/L	
Urea	5.3	2.5-7.8 mmol/L	
Creatinine	64	59-104 µmol/L	
Glomerular filtration rate	>90	90-200 mL/min	
Magnesium	1.01	0.70-1.0 mmol/L	
Creatine kinase	29	40-320 U/L	
Bilirubin	6	0-21 µmol/L	
Alanine aminotransferase	11	0-41 U/L	
Alkaline phosphatase	118	30-130 U/L	
Gamma-glutamyl transferase	74	10-71 U/L	
Total protein	53	60-80 g/L	
Albumin	18	35-50 g/L	
Globulin	35	20-40 g/L	
Ig G	6.25	7-16 g/L	
Ig A	3.94	0.7-4.0 g/L	
Ig M	1.09	0.4-2.3 g/L	
Adjusted calcium	2.18	2.20-2.60 mmol/L	
Phosphate	0.26	0.80-1.50 mmol/L	
C3	2.23	0.90-1.80 g/L	
C4	0.26	0.10-0.40 g/L	
Rheumatoid factor	20	0-14 IU/mL	
CCP antibody	8	0-17 U/mL	
ANA	Negative	Negative	
ENA antibody screen	Negative	Negative	
Double-stranded DNA antibody	<9.8	0-27 IU/mL	
ANCA	Negative	Negative	
Myeloperoxidase Ab	<3.2	0-20 U	
Proteinase-3 Ab	<2.4	0-20 U	
International normalized ratio	1.6	2.0-4.0	
Prothrombin time	18.7	10-13	
Activated partial thromboplastin time (APTT)	42.9	22.5-28.0	
APTT ratio	1.7	-	
Prostate-specific antigen	7.1	0-6.5 µg/L	

Radiology summary

Chest Imaging

Chest X-ray (CXR; anteroposterior, erect): Inspiratory effort was limited; the heart and mediastinum were poorly assessed. Right basal atelectatic changes were noted, likely post-inflammatory. There was an unchanged blunting of the right costophrenic angle. No evidence of acute consolidation, pneumothorax, or left pleural effusion was identified.

Repeat CXR (posteroanterior, erect, obtained two weeks later): Heart size was at the upper limit of normal; the mediastinum was normal. There was persistent right lower zone linear atelectasis, in keeping with post-inflammatory change. No acute collapse, consolidation, effusion, or pneumothorax was noted.

Abdominal and pelvic imaging

CT of the Abdomen and Pelvis

Bilateral lower lobe subsegmental consolidations were noted (suggestive of infection or inflammation). Inflammatory changes/thickening of retroperitoneal fascial planes were visible, most prominent in the left perinephric region. There was thickening of the urinary bladder wall, and the prostate was enlarged, elevating the bladder base. Uncomplicated diverticular disease of the colon was noted. There was no evidence of abscess, obstruction, or free fluid. Degenerative changes were present in the spine, and a large benign bone island was seen in the right iliac bone.

Impression

There was retroperitoneal inflammation, most marked around the left perinephric area, suggesting possible retroperitoneal fibrosis or a perinephric inflammatory process. No abscess or intra-abdominal collection was identified.

MRI of the Abdomen

The MRI confirmed inflammatory changes along retroperitoneal fascial planes, especially in the left perinephric and periureteric regions. No new collections were noted.

Spine and musculoskeletal imaging

MRI of the Lumbar Spine and Pelvis

There was multilevel degenerative disc and facet joint disease, with canal and foraminal stenosis at the L3-L4, L4-L5, and L5-S1 levels. Pelvic myositis and associated soft tissue edema were present. Red marrow expansion was noted, which may have reflected a chronic inflammatory or anemic state.

X-rays of the knees and hands

**Figure 1:**
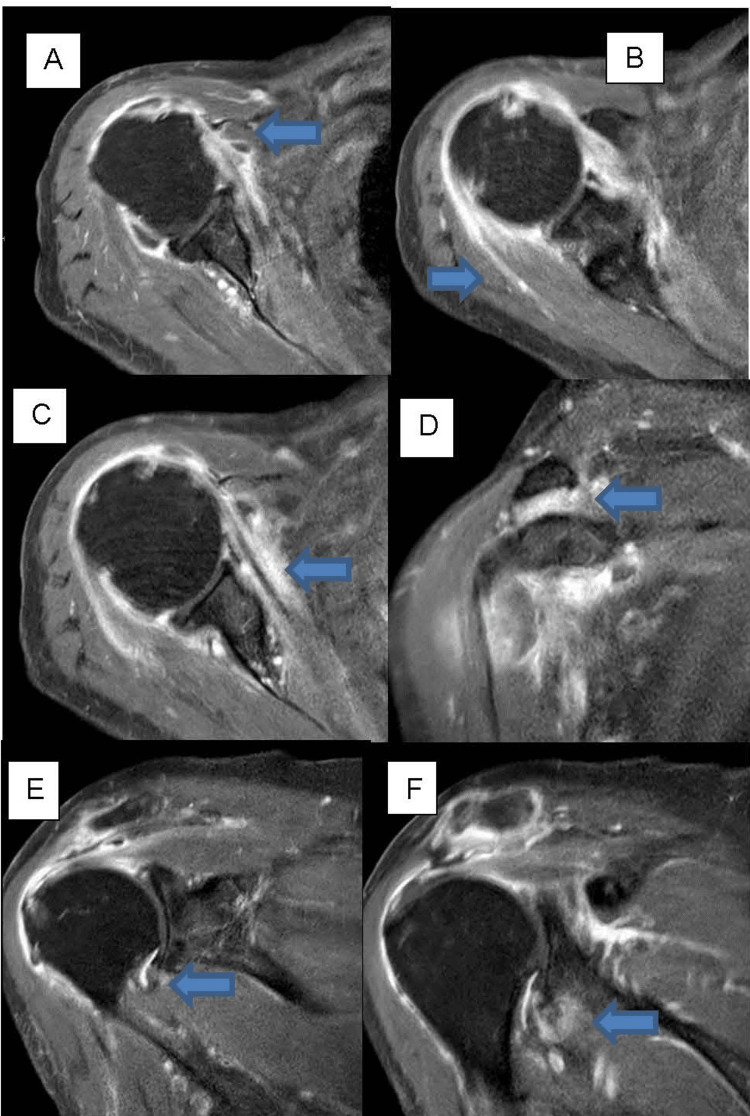
MRI of the shoulder Axial and coronal short tau inversion recovery (STIR) images display inflammation at non-synovial/periarticular sites (blue arrows). (A) Tendon origins of the short head of the biceps brachii and coracobrachialis muscles; (B) Intramuscular tendon of the infraspinatus muscle; (C) Intramuscular tendon of the subscapularis muscle; (D) Coracoclavicular ligament; (E) Axillary recess of the fibrous humeroglenoidal joint capsule; (F) Tendon origin of the long head of the triceps brachii muscle.

**Figure 2:**
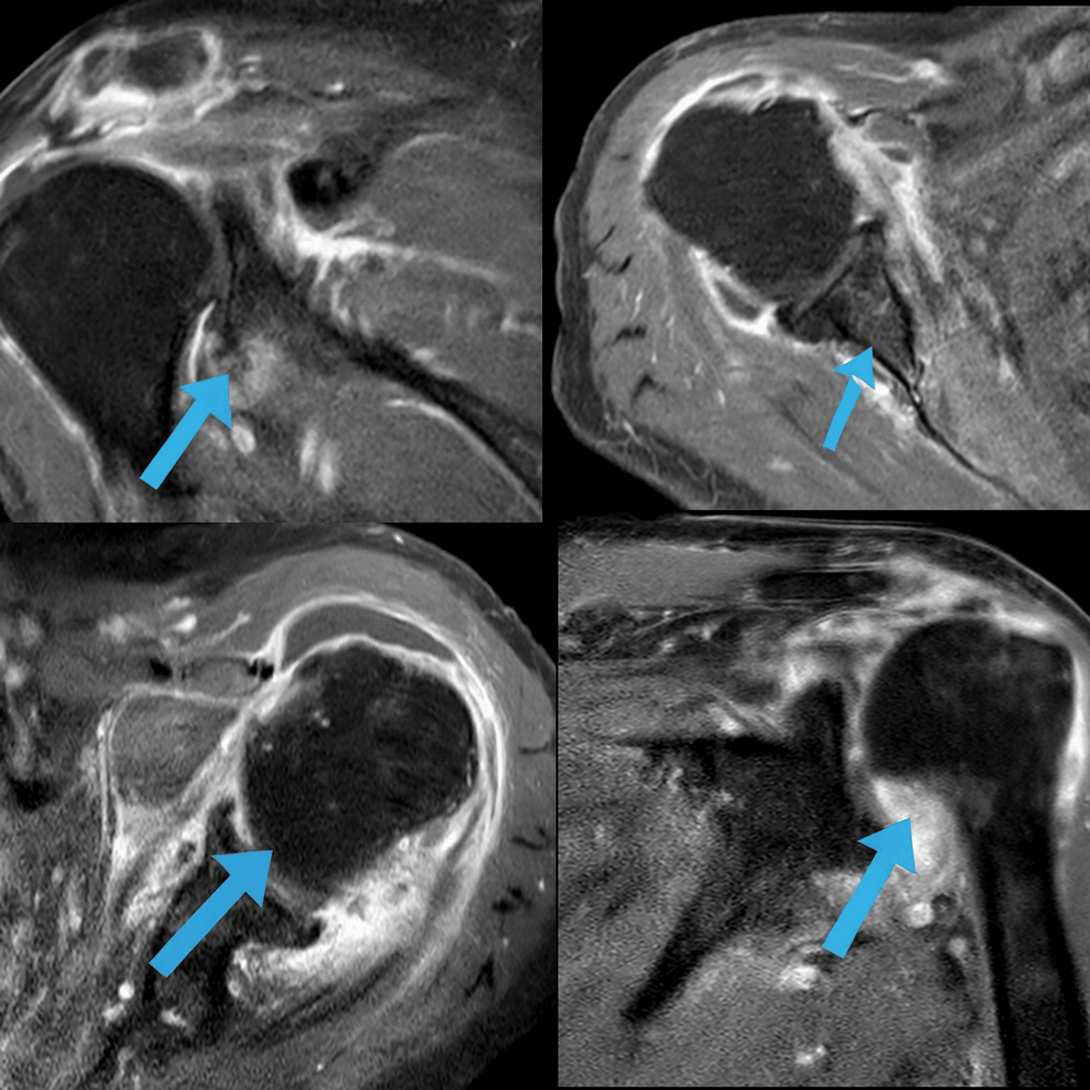
MRI of both shoulders Axial and coronal short tau inversion recovery (STIR) images display bilateral synovitis, bursitis, capsulitis, and peritendinitis characteristic of PMR, with no evidence of focal destructive lesions or infective processes.

**Figure 3:**
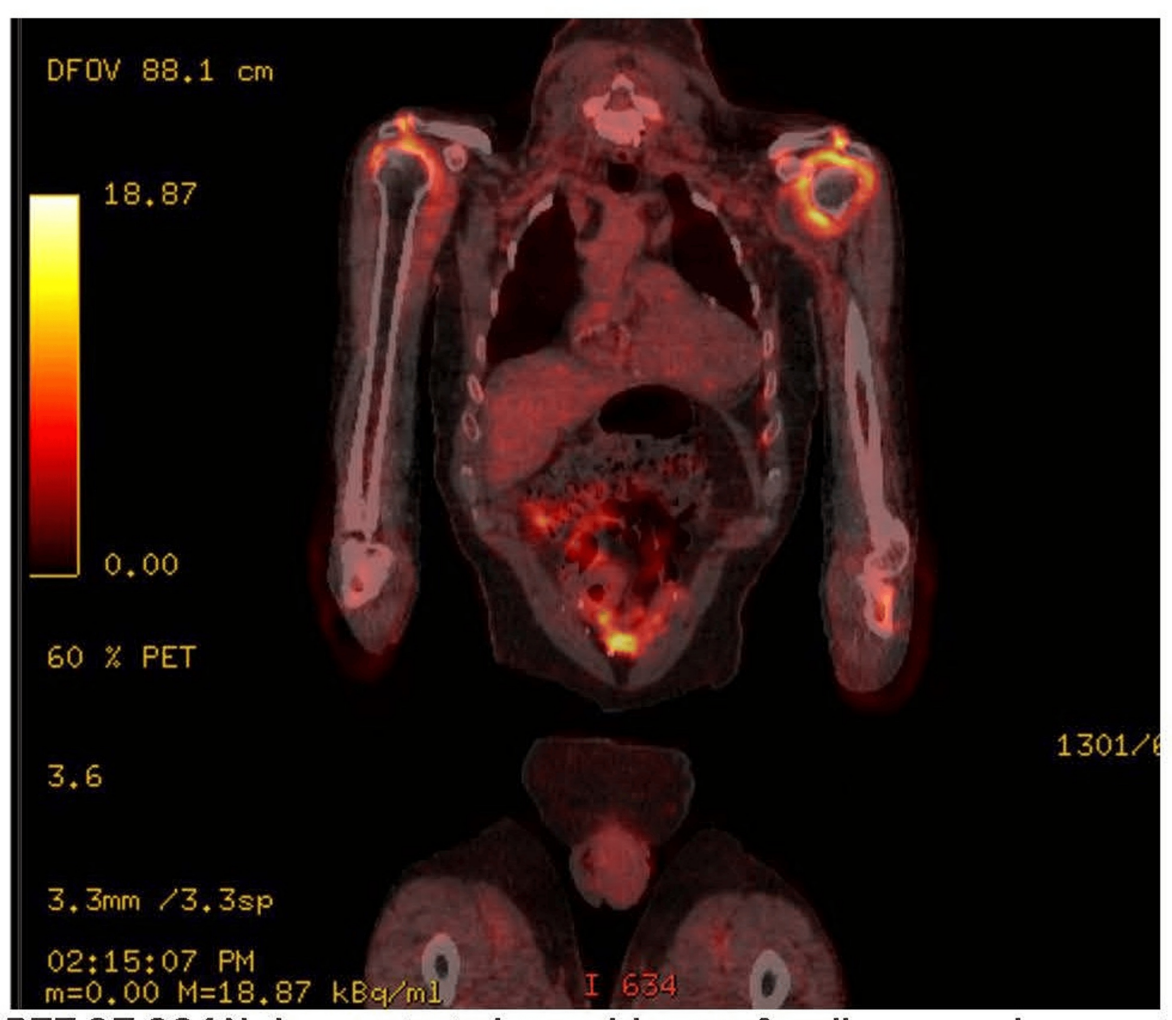
The PET CT demonstrated extensive symmetric periarticular fluorodeoxyglucose activity indicative of an active inflammatory process, with no evidence of focal destructive lesions, deep-seated infection, or vasculitis.

Osteoarthritic changes were noted. As part of the diagnostic evaluation, a CT scan of the abdomen and pelvis demonstrated no evidence of infection or malignancy. Given the patient’s persistent shoulder pain and markedly raised inflammatory markers, an MRI of both shoulders was performed, revealing bilateral subacromial and subdeltoid bursitis consistent with previously reported imaging findings in PMR (Figures 1, 2) [4]. To exclude occult infection, malignancy, or large-vessel vasculitis, a PET-CT scan was subsequently obtained. PET-CT demonstrated increased fluorodeoxyglucose (FDG) uptake in the shoulders and hips consistent with bursitis, but no evidence of malignancy or active infection (Figure 3). This stepwise imaging approach provided a comprehensive anatomical and metabolic assessment, enhancing diagnostic confidence and excluding key differential diagnoses.

Based on the clinical presentation, laboratory results, and characteristic imaging features, a diagnosis of PMR was established. The patient was commenced on oral prednisolone 15 mg daily, resulting in a dramatic improvement in shoulder pain and stiffness within one week. Repeat testing demonstrated a marked decline in inflammatory markers, with CRP decreasing from >200 mg/L to 0.9 mg/L (Table 2).

**Table 2:** Follow-up results demonstrating normalization of inflammatory markers (CRP 0.9 mg/L, ESR 5 mm/hr) and improvement in hematologic parameters following prednisolone 15 mg daily

Blood Test	Test Value	Normal Value
Hemoglobin	139	132-170 g/L
White cell count	15.2	4.3-11.2 x10⁹/L
Platelets	351	150-400 x10⁹/L
Red cell count	4.52	4.29-5.69 x10¹²/L
Mean cell volume	92	81-97 fL
Haematocrit	0.416	0.387-0.492
Mean corpuscular hemoglobin	30.8	26.9-33 pg
Mean corpuscular hemoglobin concentration	334	320-359 g/L
Neutrophils	13.17	2.1-7.4 x10⁹/L
Lymphocytes	1.27	1.0-3.6 x10⁹/L
Monocytes	0.66	0.3-1.0 x10⁹/L
Eosinophils	0.06	0.02-0.5 x10⁹/L
Basophils	0.03	0.02-0.1 x10⁹/L
ESR	5	5-20 mm/h
CRP	0.9	0-5 mg/L

## Discussion

PMR remains a primarily clinical diagnosis, but the exclusion of mimicking conditions is crucial. Infections, metastatic malignancies, and inflammatory arthritis can all present with shoulder and pelvic girdle pain accompanied by elevated inflammatory markers. In our patient, inflammatory markers remained persistently elevated for several months, prompting a comprehensive diagnostic evaluation.

An MRI can demonstrate synovitis and bursitis, but lacks specificity. In contrast, PET-CT provides a whole-body metabolic overview, allowing detection of characteristic PMR patterns such as symmetric periarticular uptake in the shoulders and hips, while simultaneously ruling out occult malignancy, infection, or large-vessel vasculitis [4,5]. Although ultrasound is more readily available and cost-effective, its ability to evaluate only localized inflammation limits its diagnostic yield compared with PET-CT, which can visualize systemic inflammatory activity and extravascular uptake.

Several studies have confirmed the diagnostic value of PET-CT in PMR. Blockmans et al. demonstrated that whole-body PET could detect characteristic findings in PMR and GCA [6]. Henckaerts et al. further described distinctive FDG uptake patterns in PMR compared with controls [7]. Yamashita et al. corroborated these findings, showing metabolic evidence of both bursitis and large-vessel vasculitis [8]. Dejaco et al. highlighted the role of imaging integration in classification criteria to improve diagnostic confidence [9]. More recently, Heras-Recuero et al. (2023) proposed a SCORE 16 method to distinguish PMR in GCA overlap settings, and Serrano-Combarro et al. (2024) demonstrated that PET-CT retains diagnostic utility even in patients already receiving corticosteroids, though vascular uptake may be diminished. The 2024 PET/CT Atlas of PMR further supports PET-CT as a potential gold standard in diagnostically challenging cases [10].

In our patient, persistent elevation of CRP and ESR, combined with nonspecific MRI findings, initially raised concern for occult infection or malignancy. PET-CT was instrumental in excluding these differential diagnoses and revealed a symmetric periarticular FDG uptake pattern without nodal, visceral, or focal osseous lesions, which is characteristic of PMR and distinct from infection or metastatic disease. While PET-CT is not mandatory in all suspected cases, it provides valuable diagnostic clarity when conventional imaging, such as CT chest-abdomen-pelvis, is non-diagnostic.

This report emphasises the clinical presentation and diagnostic workup rather than the technical parameters of PET-CT acquisition. The case illustrates how integrating appropriate imaging with clinical findings can expedite diagnosis, prevent unnecessary invasive investigations, and enable timely initiation of corticosteroid therapy. Early recognition of PMR in elderly patients presenting with unexplained musculoskeletal pain and elevated inflammatory markers remains key to improving outcomes.

## Conclusions

PMR can present atypically, often mimicking infection, malignancy, or large vessel vasculitis, especially in elderly patients with constitutional symptoms and persistently elevated inflammatory markers. In this case, PET-CT played a crucial role by excluding differential diagnoses such as malignancy and vasculitis while demonstrating diffuse periarticular and interspinous FDG uptake consistent with inflammatory arthropathy. This imaging modality offered essential diagnostic clarity in an otherwise ambiguous clinical context, ultimately supporting the diagnosis of PMR despite its atypical presentation. Clinicians should maintain a high index of suspicion and consider PET-CT in similar complex cases to avoid misdiagnosis and ensure timely, appropriate management. 
